# High Throughput Manufacturing of Bacteriophages Using Continuous Stirred Tank Bioreactors Connected in Series to Ensure Optimum Host Bacteria Physiology for Phage Production

**DOI:** 10.3390/v10100537

**Published:** 2018-10-01

**Authors:** Francesco Mancuso, Jiahui Shi, Danish J. Malik

**Affiliations:** Chemical Engineering Department, Loughborough University, Loughborough LE11 3TU, UK; F.Mancuso@lboro.ac.uk (F.M.); J.Shi-16@student.lboro.ac.uk (J.S.)

**Keywords:** bacteriophages, continuous production, *E. coli*, mathematical modelling, phage T3, process intensification, synthetic medium

## Abstract

Future industrial demand for large quantities of bacteriophages e.g., for phage therapy, necessitates the development of scalable Good Manufacturing Practice compliant (cGMP) production platforms. The continuous production of high titres of *E coli* T3 phages (10^11^ PFU mL^−1^) was achieved using two continuous stirred tank bioreactors connected in series, and a third bioreactor was used as a final holding tank operated in semi-batch mode to finish the infection process. The first bioreactor allowed the steady-state propagation of host bacteria using a fully synthetic medium with glucose as the limiting substrate. Host bacterial growth was decoupled from the phage production reactor downstream of it to suppress the production of phage-resistant mutants, thereby allowing stable operation over a period of several days. The novelty of this process is that the manipulation of the host reactor dilution rates (range 0.1–0.6 hr^−1^) allows control over the physiological state of the bacterial population. This results in bacteria with considerably higher intracellular phage production capability whilst operating at high dilution rates yielding significantly higher overall phage process productivity. Using a pilot-scale chemostat system allowed optimisation of the upstream phage amplification conditions conducive for high intracellular phage production in the host bacteria. The effect of the host reactor dilution rates on the phage burst size, lag time, and adsorption rate were evaluated. The host bacterium physiology was found to influence phage burst size, thereby affecting the productivity of the overall process. Mathematical modelling of the dynamics of the process allowed parameter sensitivity evaluation and provided valuable insights into the factors affecting the phage production process. The approach presented here may be used at an industrial scale to significantly improve process control, increase productivity via process intensification, and reduce process manufacturing costs through process footprint reduction.

## 1. Introduction

The widespread inappropriate use of antibiotics in both humans (clinical medicine) and animals (livestock industry) worldwide has led to an acceleration in the emergence and global spread of multidrug antibiotic resistant bacterial clones [1]. The problem of antibiotic resistance is a complex one requiring global coordination for antibiotic stewardship to preserve the efficacy of current treatments. In much of the world outside Europe and North America, lifesaving antibiotics are sold without a prescription or oversight by health professionals [2]. In the period between 1940–1962, 20 new classes of antibiotics were introduced to the market; however, since 1962, there has been a discovery void, with only two new classes reaching this stage [3]. The speed of resistance development has been faster than the rate of discovery of new antibiotics [4]. The substantial public health threat from antibiotic resistance includes jeopardising the effectiveness of treatments in modern medicine from minor elective surgeries to cancer therapy.

Initiatives to develop new therapeutic approaches with novel mechanisms of activity against Multi-Drug Resistant (MDR) bacterial pathogens include the potential use of lytic bacteriophages [5,6]. Lytic bacteriophages (commonly referred to as phages) are viruses that infect and kill bacteria, and they represent a promising approach for the targeting of bacterial infections in a treatment known as phage therapy [7,8,9,10]. The specificity of bacteriophages and their potential role in maintaining healthy microbiota makes them an attractive alternative to employing antibiotics. Technical advances are reducing the cost, ease of processing, and sequencing times of next-generation sequencing (NGS), thereby allowing rapid culture-independent identification of disease-causing bacteria [11]. These developments increase the opportunities for using narrow spectrum of antibiotics where the infection-causing bacterial agent is known, thereby opening-up the possibility of employing phages for therapeutic purposes [12]. A number of recent studies in animals and humans have been carried out to investigate the clinical safety and therapeutic or prophylactic efficacy of phages against *Pseudomonas aeruginosa*, *Staphylococcus aureus*, *Acinetobacter baumannii, Escherichia coli*, and *Salmonella enteritidis* [13,14,15,16].

CRISPR/Cas-mediated genome engineering approaches are being developed to repurpose phages for the sequence-specific targeting of bacteria within complex bacterial populations that are capable of distinguishing between pathogenic or commensal bacterial species through targeting virulence or essential chromosomal genes [17]. CRISPR-Cas9 targeting bacterial chromosomal genes have recently been encapsulated in phage capsids by genetically encoding the machinery in phagemids (plasmid packaged in the capsid), thereby using the species-level specificity of phages to achieve CRISPR-Cas delivery [18,19].

Increasing future demand for bacteriophages in different fields including food, agriculture, veterinary, and human medicine requires the development of scalable GMP-compliant (cGMP) phage manufacturing platforms. Existing bioprocess engineering approaches that are used for the manufacture of biotherapeutics e.g., monoclonal antibodies and vaccine manufacture, may be adapted for phage production. However, there are important differences, including the relatively large size of phages (~100 nm), with implications for the development of process unit operations. Conventional chromatographic materials, for example, are currently designed to allow proteins access to the internal pore structure and large surface area that are not readily accessible to large phage particles, reducing the separation capacity of the resins [20]. Therefore, new innovations are needed such as the use of monolith-based chromatography supports to overcome such challenges [21]. The manufacture of large quantities of phages at low cost necessitates the development of continuous production techniques and a move away from batch processing in order to improve productivity and reduce the process footprint [22]. Regulatory agencies (e.g., the United States Food and Drug Administration, FDA) have strict requirements regarding controlling the product quality within specified limits for pharmaceuticals. A fundamental understanding of the underlying kinetics and the influence of processing conditions on product quality attributes is an essential prerequisite to ensure the implementation of online or at-line process control strategies as part of a Quality by Design (QbD) framework now in favour by USFDA for the manufacture of pharmaceuticals.

Phages are typically produced in batch fermenters e.g., shaken flasks, and more recently in disposable wave bags that are used for tissue cell culture; there are no real issues with regard to residence times and complex control strategies [23,24]. The downsides for industrial scale batch fermentation include higher capital costs, large process footprints, labour-intensive operation, that the proportion of downtime compared with production time can be high, a lack of process control, and variability of product quality [24]. The continuous upstream production of phages using chemostat systems has heretofore received little attention in the published literature, which instead has focused on using such systems for studying coevolution processes [25,26,27]. Decoupling the bacterial host propagation from phage production removes the selection pressure for bacteria mutation and should allow stable long-term steady-state operation of the process [28]. Semi-continuous production approaches using such a strategy include a two-stage self-cycling process for the production of phages [24,29,30]. Synchronous host populations at high cell concentrations are produced in the first reactor operated in batch mode. These are then passed on for infection with phages in a second batch reactor using a relatively simple control strategy based on the host population approaching a near-stationary phase prior to half of the fermentation volume being transferred to the second reactor for infection. Fresh nutrients are subsequently added to the first reactor to continue host cell propagation in the exponential growth phase, whilst the second infection vessel amplifies the phages prior to the process being repeated in a cyclic fashion.

Continuous processes are better suited to increase volumetric productivity. Using two continuous stirred tank bioreactors connected in series would allow the propagation of host bacteria at steady-state concentrations with better control over the host cell metabolic state, ensuring that the cells are optimally susceptible to phage infection in the second bioreactor. The residence times of the two bioreactors could be independently controlled by using different reactor volumes, even though the same flow rate is maintained through them. This would enable longer residence times in the host bacteria-culturing chemostat, resulting in high concentrations of bacteria in the early to middle exponential growth phase. It would also create short residence times in the phage infection bioreactor, resulting in a low MOI (multiplicity of infection) and allowing a higher number of phage replication cycles and further phage amplification in a holding tank (reactor 3) downstream of the second bioreactor. A minimum of two holding tanks may enable the continuous operation of the process. Whilst one vessel is being filled with material from the second bioreactor, the other with phage amplification completed would be emptied, cleaned, and made ready to be brought back into service.

The aim of the present work was to demonstrate the key parameters that need to be evaluated in order to characterise and hence develop an upstream continuous production process for phages as described above. An obligately lytic *E. coli* phage T3 (belonging to the *Podoviridae* family, ATCC11303-B3) and bacterial host *E. coli* (ATCC 11303) were used for the study. Bacteria host growth parameters were evaluated and fitted with Monod kinetics and phage growth parameters (adsorption constant, burst size, and latency period) were evaluated using synthetic media (glucose as the limiting substrate) in order to develop a mathematical model simulating the key features of the production process. The influence of residence time (by controlling the dilution rate) on the bacteria metabolic state and phage productivity in the second bioreactor were investigated. We show through experiments and modelling that the stable continuous manufacture of high titres of phages and process development can be readily achieved through the characterisation of a few key process parameters.

## 2. Materials and Methods

### 2.1. Strains and Media

Host bacterium *E. coli* (ATCC 11303) and its lytic phage T3 (ATCC11303-B3), belonging to the *Podoviridae* family, were sourced from LGC standards (Teddington, Middlesex, United Kingdom (UK)). A defined synthetic medium previously used by Li et al. (2011) [31] was used for bacterial growth and phage production; only the carbon source was changed to glucose at a working concentration of 2.94 gL^−1^ (16 mM). Briefly, all of the salts were prepared separately and mixed together, followed by sterilization at 121 °C: working concentration of KH_2_PO_4_ (13.3 g L^−1^), MgSO_4_ (0.586 g L^−1^), (NH_4_)_2_HPO_4_ (4 g L^−1^), citric acid (1.5542 g L^−1^), and Fe(III) citrate (0.1008 g L^−1^), with added trace elements at concentrations (details provided as Supplementary Information, Table S1). Glucose was filtered using a 0.22-µm pore size in-line syringe filter (Millipore, USA) and added to the salt solution prior to use in order to avoid any caramelisation reactions in the autoclave. All of the salts and chemicals were purchased from Fisher Scientific UK. Starter cultures were prepared using lysogeny broth (LB Miller, Fisher Scientific, Loughborough, Leicestershire, UK) and LB plates (LB broth Miller, Fisher Scientific and 1.5 % Microbiological Agar, Oxoid, Basingstoke, Hampshire, UK).

### 2.2. Continuous Stirred Tank Bioreactors

**Figure 1:**
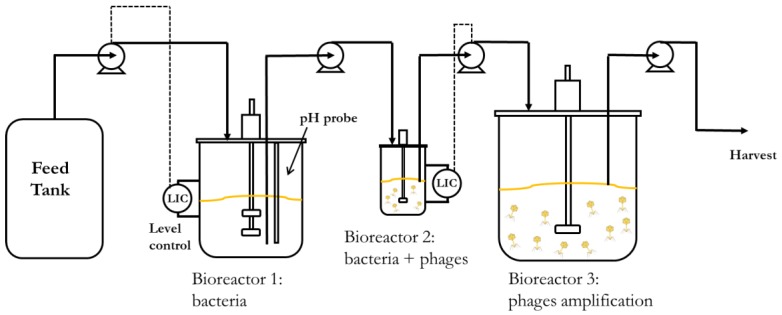
Schematic showing layout of the continuous phage production process, which was a three-stage continuous stirred tank system with bioreactors connected in series: the first reactor (Bioreactor 1, R1) had a fixed working volume of 500 mL, the second reactor (Bioreactor 2, R2) had variable working volumes ranging from 33 mL to 100 mL, thereby permitting different dilution rates (D_2_) for phage production using the same flow rate through the process, and the third reactor (Bioreactor 3, R3) was operated as a semi-batch reactor. Host *E. coli* were propagated in R1; the working volume was controlled using a level control system. The T3 infection of bacteria occurred in R2, which was fed with host bacteria continuously from R1. R2 was operated below the dilution rate where phage washout would occur; hence, steady-state operation allowed the phage population to be continuously maintained in R2. Phage-infected cells and free-floating phage were continuously withdrawn from R2 to R3 (flow rate synchronized with R1 using a level control system on R2) where multiple cycles of infection resulted in further phage amplification and completion of the phage production process.

All of the experiments were conducted using a Biostat B (Sartorius Stedim Plastics GmbH, Goettingen, Germany) employing a 1-L water-cooled jacketed glass bioreactor. The host bacterium propagation reactor (R1) working volume was 0.5 L; bacteria were grown in batch mode at 37 °C for three hours prior to the start-up of continuous operation. Calibrated peristaltic pumps (101 U/R, Watson Marlow) were used for the process. A pump was used to withdraw host bacteria containing solution from R1 and transfer it at a constant rate to bioreactor 2 (R2) (Figure 1). The level in R1 was kept constant by continuously supplying fresh substrate from the substrate feed tank (volume 10 L) using a level controller actuating a peristaltic pump. This ensured that the volume in R1 was kept constant. Following the attainment of steady-state conditions in R1 at a given dilution rate D_1_ (hr^−1^) (this typically took ~6 hr), R2 was inoculated with phages at an initial inoculum of (10^8^ PFU). A third pump was switched on to transfer bacteria and phages to the semi-batch reactor (R3) acting as a holding tank to complete phage amplification. Experimental runs were carried out at different dilution rates of R1 and different working volumes of R2 (details provided as Supplementary Information, Table S2). Optical density at 600 nm was monitored at-line using a spectrophotometer (Shimadzu UV mini 1240, Milton Keynes, Buckinghamshire, UK); pH and temperature were continuously monitored with probes inside R1.

The dilution rates that were used for R1 (D_1_) were 0.1 hr^−1^, 0.2 hr^−1^, 0.3 hr^−1^, 0.4 hr^−1^, 0.5 hr^−1^, and 0.6 hr^−1^, and these were changed using a peristaltic pump that ensured flow rates between 50–300 mL hr^−1^. Flow rates once set were kept constant; the dilution rate of reactor 2 (R2) was adjusted by changing the working volume of the reactor. The dilution rates that were tested in the second reactor were 3 hr^−1^, 4 hr^−1^, and 6 hr^−1^, and the volume of the third reactor allowed the collection of output from R2 for periods between 5–10 hrs (Table S2).

Bacteria were aseptically sampled from the outlet flow of R1 and phages from the outlet flow of R2. In R3, samples were monitored every hour for a period of at least four hours, and then after overnight running of the process for a period of typically ~16 hr. Colony Forming Units (CFU) counts were performed using spot tests of 10 µL of different dilutions of the sample on LB plates. Each dilution was spotted four times, and the average was used. Plaque Forming Units (PFU) counting was performed using the double-layer method [32] and spotting four times with 10 µL of the phage-containing samples. A second layer composed of 15 mL of LB agar (with a concentration of agar at 0.8 wt%) containing 50 μL of centrifuged overnight grown *E. coli* host was added on top of the LB plates and left to dry before spotting.

Both bacteria and phage plates were incubated overnight at 37 °C, and colonies or plaques were counted after 16 hrs of incubation. Each measurement was repeated three times, and the values presented are the average values from the spot tests ± SD.

The continuous phage production process that was used here was found to be stable, and could be operated without interruption for several days (typically a week), after which the process was taken offline for cleaning and re-sterilisation, which could be done quickly using CIP (cleaning-in-place) approaches. The process was found to be easy to start up, steady-state was achieved within six hours.

### 2.3. Latent Period, Burst Size, and Adsorption Constant Determination

Latent period and burst sizes for host propagation reactor dilution rates 0.4 hr^−1^, 0.5 hr^−1^, and 0.6 hr^−1^ were determined by performing one-step growth according to a standard published protocol by Hyman and Abedon [33]. Briefly, 1 mL of steady-state *E. coli* culture collected aseptically from the reactor was transferred to a 1.5-mL Eppendorf tube. Prewarmed phage solution (37 °C) at a known concentration was added to achieve a multiplicity of infection (MOI) of 0.1. The mixture was gently vortexed and incubated for 5 min at 37 °C without agitation. After 5 min, unadsorbed phages were removed by centrifugation (10,000 *g* for 2 min), supernatant discarded and infected cells were re-suspended and washed twice in 1 mL of SM buffer (Trizma base (50 mM, Sigma Aldrich, Gillingham, Dorset, UK), NaCl (100 mM, Fisher Scientific), MgSO_4_·7H_2_O (8 mM, Fisher Scientific), 5M HCl (~10 mL added per litre to adjust pH to pH 7.5)) each time to remove reversibly bound phages with centrifugation steps following each wash step. Infected cells were re-suspended in nine mL of fresh supernatant from the host bacteria reactor R1 (with cells removed by centrifugation and filtration of media through a 0.2-µm Whatman filter) and incubated at 37 °C with agitation. The number of infection centres was evaluated by diluting a 10-µL aliquot at time zero, and spotted using the double-layer method. Samples were taken at regular intervals and spotted using the double-layer method. Burst size was calculated based on the number of phages released per infected cell. The difference in supernatant phage concentration at the beginning and end of the phage replication cycle provided the number of phages formed.

The adsorption constant for host propagation reactor dilution rates of 0.4 hr^−1^, 0.5 hr^−1^, and 0.6 hr^−1^ were determined according to a standard published protocol by Hyman and Abedon [33]. Briefly, 1 mL of steady-state *E. coli* culture that was collected aseptically from the reactor was transferred to a 1.5-mL Eppendorf tube. Prewarmed phage solution at the same temperature (37 °C) was added to the bacteria culture to achieve a multiplicity of infection (MOI) of 0.1, gently vortexed to mix, and incubated at 37 °C without agitation. Then, 50-µL samples were withdrawn every minute and transferred to new tubes containing 950 µL of SM buffer with a few drops of chloroform to lyse cells and release a reversibly bound phage. After gentle mixing, the tube was incubated over ice to slow down the binding and further adsorption. Samples were diluted and spotted using the double-layer method. Controls in the absence of bacteria provided the starting concentration of phages. The initial concentration of bacteria was determined separately in triplicates. The adsorption constant was calculated assuming first-order kinetics and from the slope of the logarithm of free phages versus the time and initial concentration of bacteria.

### 2.4. Mathematical Modelling

#### Modelling the Two Continuous Stirred Tank Bioreactors Operated in Series

Underlying assumptions in building the mathematical model included that the phages were obligately lytic phages that infect the bacterial host and quickly produce and release progeny, resulting in a sudden lethal burst (or lysis) of the host cell. The adsorption of phage particles to the host bacterium was considered to follow the law of mass action i.e., a first-order process with respect to the concentration of phage (P, PFU L^−1^) and concentration of bacterial cells (C, CFU L^−1^). Following adsorption, the injection of phage DNA results in phage gene expression and phage protein synthesis; a number of phage progeny (b = burst size) are matured within the cell until lysis occurs after a certain lysis or lag time (L, hr). The adsorption rate constant (δ, L hr^−1^) is the parameter determining how rapidly the phage adsorbs to the bacterium. Experimentally determined values for *E. coli* phage T3 burst size (b = 10–40, Figure S1) and lag time (~10 min, Figure S1) were used for model simulations. In situ phage amplification measurements for R2 were fitted using the mass action law and gave a best fit value of δ ~3.6 × 10^−11^ L hr^−1^, which was used for mathematical modelling the data for R2. The experimentally determined adsorption rate values varied with dilution rate D_1_ in the range (1.8 × 10^−9^–9 × 10^−9^ L hr^−1^, Figure S2). The typical adsorption rates of virulent phages are in the range of 10^−10^ to 10^−9^ L hr^−1^ [34].

The two-stage chemostat system had a cell-only bioreactor (reactor 1, R1), which provided a continuous supply of high-density susceptible cells in the early to mid-growth phase to a phage propagation reactor (reactor 2, R2) connected in series (Figure 1). R1 was operated with fresh nutrient feed (synthetic medium with glucose as the limiting substrate, S_o_, g L^−1^) that was fed to reactor 1 (flow rate, q, L hr^−1^); host bacterial cells were maintained in the log growth phase at steady-state (cell concentration in R1, C_1_, CFU L^−1^), and removed at the same flow rate. The metabolic state of the host bacteria and substrate utilisation in R1 was governed by the residence time in reactor 1 (V_1_/q, where V_1_ is the volume of fermentation broth in R1), which was controlled by regulating the dilution rate (D_1_ = q/V_1_, hr^−1^). The steady-state output from R1 was fed to R2, where bacteria were continuously infected by the free-floating phage population (P_2_) that was resident in bioreactor R2. The steady-state bacterial population in reactor 2 (C_2_) and phage population P_2_ were controlled by regulating the dilution rate in reactor 2 (D_2_ = q/V_2_, hr^−1^) through maintaining a fixed volume in R2 using a level control system. The phage product stream at flow rate q was continuously withdrawn out of R2. The phage production rate was simply P_2_D_2_ (PFU L^−1^ hr^−1^).

Balances around R1

Assumptions: Ignore cell death and substrate utilisation for cell maintenance.

Cell balance:

(1) $$\;r_{1}V_{1}=qC_{1}\;$$

where r_1_ (CFU L^−1^ hr^−1^) is the host cell growth rate in reactor R1, V_1_ (l) is the volume of the mixture in reactor R1, q is the volumetric flow rate (L hr^−1^) through reactor R1, and C_1_ (CFU L^−1^) is the steady-state host cell concentration in reactor R1.

Substrate balance:

(2) $$\;qS_{o}-Y_{S/C}r_{1}V_{1}=qS_{1}\;$$

where Y_S/C_ is the substrate yield coefficient (g substrate per CFU cells), and S_o_ and S_1_ (g L^−1^) are the reactor R1 inlet and outlet limiting substrate (glucose) concentrations, respectively.

Host growth kinetics were modelled using the Monod equation:

(3) $$r_{1}=\frac{\mu_{m}C_{1}S_{1}}{K_{m}+S_{1}}\;$$

The Monod equation was found to fit the exponential growth experimental data, and the fitted parameter values were evaluated. µ_m_ is the maximum specific growth rate (1 hr^−1^), and K_m_ is the Monod constant (1.5 g L^−1^).

Balances around R2:

Assumptions: Ignore cell death, the substrate utilisation for cell maintenance, and any phage inactivation in the reactor.

Cell balance:

(4) $$\;qC_{1}+r_{2}V_{2}-\delta C_{2}P_{2}V_{2}=qC_{2}\;$$

where r_2_ (CFU L^−1^ hr^−1^) is the host cell growth rate in reactor R2, δ (L hr^−1^) is the phage adsorption rate constant, P_2_ (PFU l^−1^) the free-floating phage population, and C_2_ (CFU L^−1^) is the steady-state host cell concentration in reactor R2.

Substrate balance:

(5) $$\;qS_{1}-Y_{S/C}r_{2}V_{2}=qS_{2}\;$$

where S_2_ (g L^−1^) is the glucose concentration in R2.

Phage balance:

(6) $$\;-\delta C_{2}P_{2}+\delta be^{-D_{2}L}C_{2L}P_{2L}=qP_{2}\;$$

where L (hr) is the lag time, b is the phage burst size, and D_2_ is the dilution rate in R2. Although the concentrations in the phage generation term refer to those at time L min in the past, at steady-state reactor operation, these are no different to the steady-state values present in the reactor. Hence, Equation (6) may be re-written as:

(7) $$\;\delta C_{2}P_{2}\left(b^{\prime }\right)=qP_{2}\;$$

where:

(8) $$\;b^{\prime }=\left(be^{-D_{2}L}-1\right)\;$$

At steady-state combining Equations (4)–(6) and Equation (8) and solving for the phage concentration in R2 gives the following relationship:

(9) $$\;P_{2}=b^{\prime }\left(C_{1}-\frac{D_{2}}{\delta b^{\prime }}\right)+\frac{\mu_{m}}{\delta }\left(\frac{S_{2}}{K_{m}+S_{2}}\right)\;$$

Modelling Reactor 3 as a semi-batch reactor:

Cell balance:

(10) $$\;qC_{2}+r_{3}V_{3}-\delta C_{3}P_{3}V_{3}=\frac{dC_{3}V_{3}}{dt}\;$$

where r_3_ (CFU L^−1^ hr^−1^) is the host cell growth rate in reactor R3, P_3_ (PFU l^−1^) is the free-floating phage population, and C_3_ (CFU L^−1^) is the host cell concentration in reactor R3 and volume of reactor mixture V_3_ (l) varying with time due to flow q into the vessel.

Substrate balance:

(11) $$\;qS_{2}-Y_{S/C}r_{3}V_{3}=\frac{dS_{3}V_{3}}{dt}\;$$

where S_3_ (g L^−1^) is the glucose concentration in R3.

Phage balance:

(12) $$\;qP_{2}-\delta C_{3}P_{3}V_{3}+b\delta V_{3}C_{3L}P_{3L}=\frac{dP_{3}V_{3}}{dt}\;$$

Model simulations:

The numerical integration of delay differential equations was carried out using dde23 in MATLAB.

Productivity of the reactor:

The productivity of R2 was assessed using the formula Productivity = D_2_ × P_2_, where D_2_ was the dilution rate, and P_2_ was the phage concentration exiting R2 or the final concentration of phages in R3.

Glucose conversion:

The glucose concentration in the three reactors was measured using a GL6 glucose analyser (Analox Instruments, Stourbridge, Worcestershire, UK) following the instructions from the manufacturer. A calibration curve was made using glucose standards, and the output of the instrument was proportional to the glucose concentration. The measured concentrations of glucose in the bioreactors were calculated based on relating the instrument output and reference to the calibration linear curve. Instrument linear sensitivity was as low as 0.125 mM of glucose. At concentrations above 25 mM, samples were diluted in deionized water. Samples were centrifuged for 3 min at 10,000 *g*, 4 °C, and the supernatant was tested for the presence of glucose. A sample of 10 µL was sufficient to assess the amount of glucose in the relevant bioreactor.

Glucose conversion, i.e., the amount of glucose that was consumed to produce new bacterial cells at a given time point was calculated using the following relationship: (C_0_ − C_x_)/C_0_, where C_0_ was the initial glucose concentration in the feed medium, and C_x_ was the concentration in the sample at the given time point.

## 3. Results

**Figure 2:**
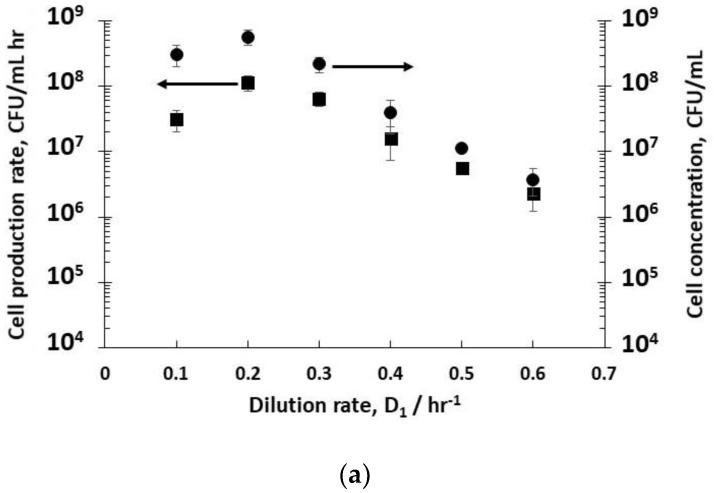

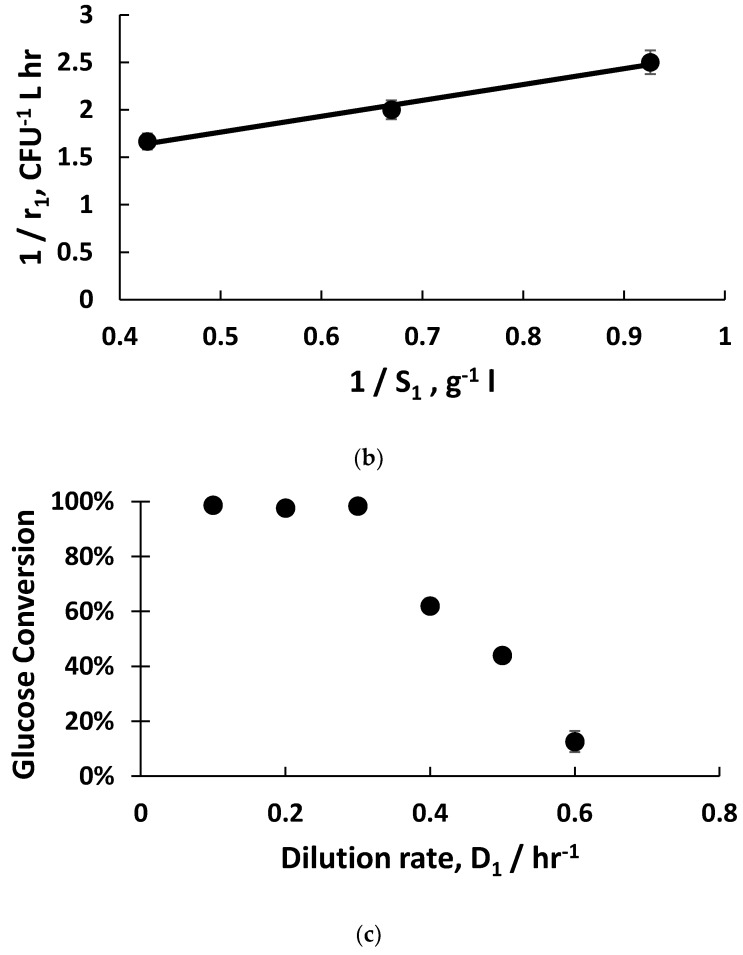
(**a**) Filled circles (●) represent the concentration of host bacterial cells in R1, and filled squares (■) represent the host bacterial cell productivity in reactor 1 (R1), which are plotted as a function of different dilution rates in R1. (**b**) Host growth rate data fitted with Monod kinetics (3) linearized as a Lineweaver–Burk plot; the slope of the line is K_m_/µ_m_, the y-intercept is 1/ µ_m_, the linear least squares fit yielded values of K_m_ (1.5 g L^−1^) and µ_m_ (1 hr^−1^). (**c**) Glucose conversion as a function of dilution rates in R1. The black filled circles (●) represent the percentage of glucose (compared with the inlet substrate concentration to R1) consumed by *E. coli* to produce new cells. The residence time in R1 was controlled using the dilution rate D_1_. Error bars represent one standard deviation.

Dilution rates (D_1_) from 0.1 hr^−1^ (residence time 10 hr) to 0.6 hr^−1^ (residence time 1.6 hr) resulted in steady-state concentrations of host bacteria at different stages in their growth phase in reactor R1 (Figure 2a). The fitting of exponential bacterial growth rates employing Monod kinetics (Equation (3)) permitted the estimation of the growth parameters (Figure 2b) with glucose as the limiting substrate; these values were subsequently used for the mathematical modelling of host bacterial growth kinetics (K_m_ = 1.5 g L^−1^; µ_m_ = 1 hr^−1^). The substrate yield coefficient was also evaluated using these data (Y_S/C_ = 1 × 10^−10^ g glucose consumed CFU^−1^). At low dilution rates (between 0.1–0.3 hr^−1^), the bacteria were in the late-stage growth phase tending to the stationary phase, and had consumed almost all of the nutrient sugar (Figure 2c), resulting in high viable host cell numbers (~10^8^ CFU mL^−1^) leaving reactor R1. Host cell productivity in R1 attained a maximum value of ~1 × 10^8^ CFU mL^−1^ h^−1^ at a dilution rate of 0.2 hr^−1^. Increasing the dilution rates to 0.4–0.6 hr^−1^ resulted in a much lower sugar consumption (between 13–50% conversion, Figure 2c), and bacteria in the early to mid- exponential growth phase with cell concentrations falling from ~6 × 10^8^ CFU mL^−1^ to ~4 × 10^6^ CFU mL^−1^ (Figure 2a). At a dilution rate of 0.5 hr^−1^, significant residual glucose concentration was detected in the R1 reactor outlet (~ 44% glucose conversion, Figure 2c), which was indicative of host bacteria in the mid-exponential growth phase.

**Figure 3:**
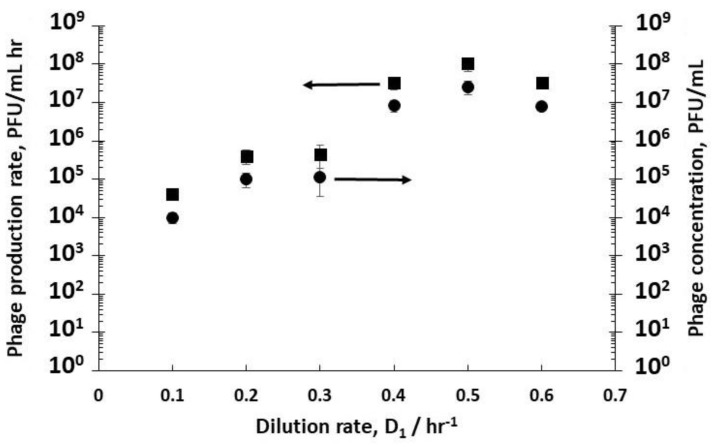
Phage productivity (■) in reactor 2 (R2) as a function of the dilution rate (D_1_) in reactor 1 (R1). The dilution rate in R2 (D_2_) was kept constant at a set value of 4 hr^−1^. The filled circles (●) show the concentration of phages (PFU/ml) in R2 at steady-state operation. Error bars represent one standard deviation.

The effect of varying D_1_ (and therefore the cell growth conditions discussed above) on phage titres in R2 was evaluated by keeping the dilution rate D_2_ in R2 constant at 4 hr^−1^ (i.e., a residence time of 15 min). Although this dilution rate value was quite high, it was below the measured phage washout rate, which occurred at D_2_ > 6 hr^−1^. Phages T3 were measured to have a relatively short lag time to burst, ~10 min (see Supplementary Figure S1). Low phage concentrations were measured leaving R2 at low dilution rates e.g., ~1.1 × 10^5^ PFU mL^−1^ at a dilution rate D_1_ of 0.3 hr^−1^ (Figure 3), whereas at dilution rates of 0.4 hr^−1^, 0.5 hr^−1^, and 0.6 hr^−1^, phage titres increased significantly to ~10^7^ PFU mL^−1^, resulting in considerably higher phage productivity, from ~10^4^ PFU mL^−1^ h^−1^ at a dilution rate of 0.1 hr^−1^ to ~10^8^ PFU mL^−1^ h^−1^ at a D_1_ value of 0.5 hr^−1^ (Figure 3).

**Figure 4:**
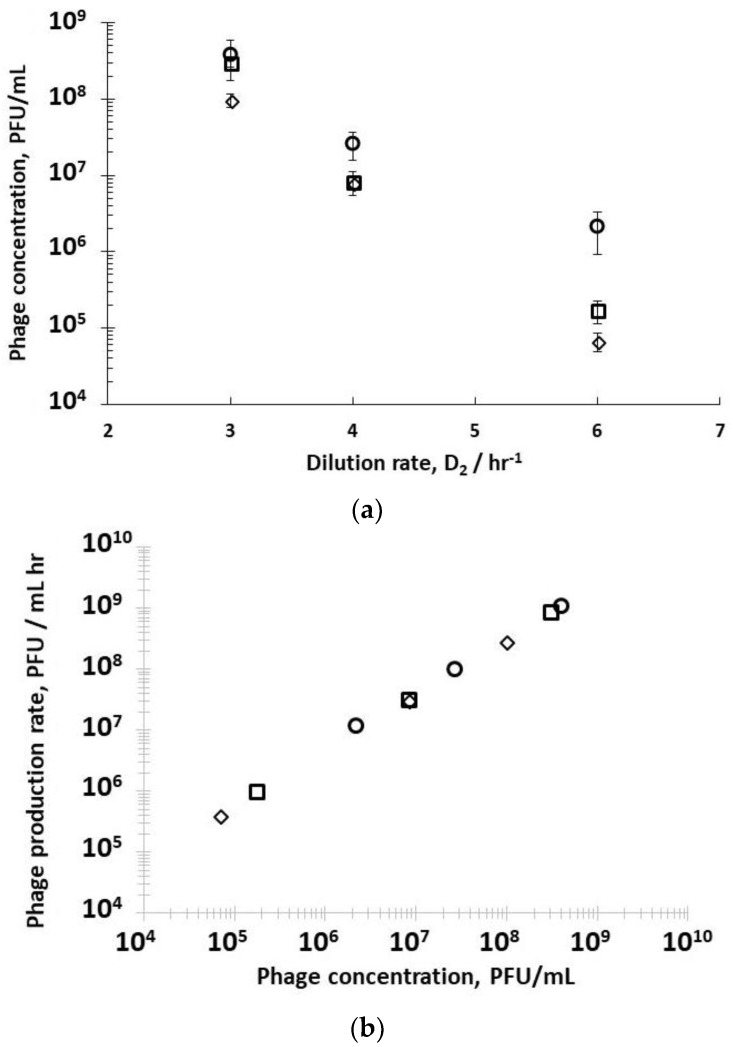
(**a**) The effect of bacteria physiology and dilution rate D_2_ on phage titres in the second reactor R2. (**b**) Phage production rate as a function of phage concentration in R2. Open squares (□) correspond to phages produced in R2 when R1 was operated at D_1_= 0.4 hr^−1^; open round circles (o) correspond to phages produced in R2 when R1 was operated at D_1_= 0.5 hr^−1^; open diamonds (◊) correspond to phages produced in R2 when R1 was operated at D_1_= 0.6 hr^−1^. Error bars represent one standard deviation.

A second set of experiments was performed to study the effect of dilution rate D_2_ on phage production using three different fixed R1 dilution rates D_1_ (0.4 hr^−1^, 0.5 hr^−1^, and 0.6 hr^−1^); this allowed control over host bacteria growth in the early (D_1_ 0.6 hr^−1^) to mid-logarithmic (D_1_ 0.4 hr^−1^ and 0.5 hr^−1^) growth phase to be fed to R2. The D_2_ values that were used were subsequently varied with values set at 3 hr^−1^, 4 hr^−1^, and 6 hr^−1^ corresponding to residence times in R2 of 20 min, 15 min, and 10 min, respectively (see Supplementary Information, Table S2). Decreasing the residence time in R2 resulted in less time for phage amplification, and concomitantly a decrease in the corresponding phage titres exiting R2 (Figure 4a) under all three set values of R1 dilution rates (D_1_ 0.4 hr^−1^, 0.5 hr^−1^, or 0.6 hr^−1^). Phage titres were higher for runs where bacteria were in the mid-log growth phase with significant differences observed at the higher dilution rate of D_2_ = 6 hr^−1^ (Figure 4a). The phage titre decreased significantly with increasing D_2_; however, phages were not washed out, despite the short residence times in R2 of 10 min at D_2_ 6 hr^−1^. At D_2_ 6 hr^−1^, significant differences in phage production rates were measured, depending on bacteria host physiology (Figure 4a). Phage production rates (P_2_ × D_2_) that were plotted as a function of phage titres P_2_ showed a linear correlation, indicating that phage amplification is consistent with the mass action law.

**Figure 5:**
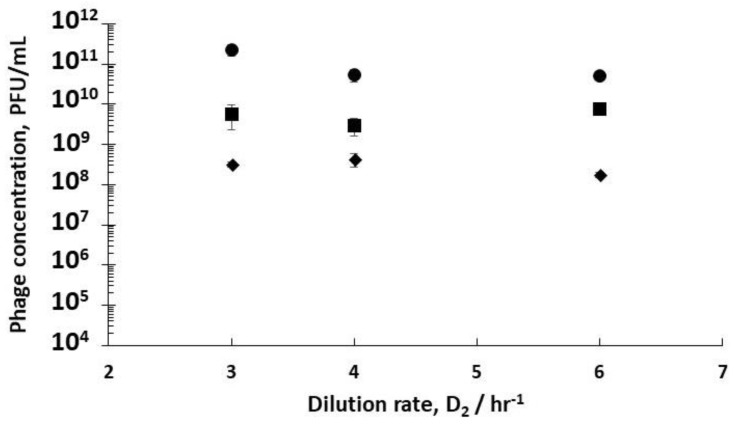
Final phage titres in Reactor 3 (R3) after overnight amplification following infection in R2 using dilution rates (D_2_). Filled squares (■) correspond to phages infected in R2 when R1 was operated at D_1_= 0.4 hr^−1^; filled round circles (●) correspond to phages infected in R2 when R1 was operated at D_1_= 0.5 hr^−1^; filled diamonds (♦) correspond to phages infected in R2 when R1 was operated at D_1_= 0.6 hr^−1^. Error bars represent one standard deviation.

Using stirred tank R3 as a holding tank operated in semi-batch mode allowed control over phage amplification with residence times ~4 hr, found to be more than enough to allow completion of the phage amplification process (see Supplementary Information, Table S3). Phage titres increased from ~10^8^ PFU mL^−1^ exiting R2 (D_2_ set at 3 hr^−1^) to ~6 × 10^9^ PFU mL^−1^ (D_1_ 0.4 hr^−1^) and ~2 × 10^11^ PFU mL^−1^ (D_1_ 0.5 hr^−1^) (Figure 5). Phage titres increased from ~9 × 10^7^ PFU mL^−1^ exiting R2 (D_2_ set at 3 hr^−1^) to ~3 × 10^8^ PFU mL^−1^ (D_1_ 0.6 hr^−1^) (Figure 5). There was a significant difference in the final R3 phage titres depending on the growth phase of the host bacteria leaving R1, which could be controlled through manipulating D_1_.

## 4. Discussion

Phages can initiate complex mechanisms depending on the physiological state of the host bacteria including lysis inhibition and pseudolysogeny, prolonging the period from infection to lysis, etc. Previous studies have demonstrated that the burst size and latency period are influenced by the host physiological state. Researchers have previously shown that the infection of slow-growing bacteria (e.g., using different media) or bacteria that have spent long residence times due to operation at low dilution rates in a chemostat resulted in a decrease in the burst size and increase in the latency period [35,36,37,38]. We have shown through using a synthetic medium with glucose as the limiting substrate that controlling the dilution rate in the host propagation reactor resulted in control over the bacterial growth rate, and in turn the physiology of the host bacterial cells (Figure 2). This allowed optimum infection conditions to be maintained in the phage infection reactor R2 (D_2_ 0.5 hr^−1^), thereby resulting in high titres of phages that could be manufactured in a continuous manner (Figure 3 and Figure 4). The experimental system allowed precise control over the residence times of bacterial host cells (in R1) and phages (in R2), allowing a steady-state analysis of phage-bacterium infection dynamics. Keeping a high dilution rate D_2_ of 6 hr^−1^ i.e., short residence times of around 10 min in R2 (Figure 4), resulted in clear differences in the phage titres leaving the reactor. This clearly showed the marked effect of the bacterial host physiology on in situ phage production dynamics. R2 was operated at a low multiplicity of infection (MOI) e.g., at the high dilution rate D_2_ of 6 hr^−1^, the MOI in R2 was between 0.01~0.2, thereby guaranteeing single infections [36]. Varying D_2_ (3 hr^−1^, 4 hr^−1^, and 6 hr^−1^) allowed different MOI values in R2, but the final achieved phage titres in R3 were not found to significantly increase with a reduction in MOI in R2 (Figure 5). As an example, using D_1_ 0.5 hr^−1^, the MOI varied from ~10 at D_2_ 3 hr^−1^ to ~1 at D_2_ 4 hr^−1^ and ~0.1 at D_2_ 6 hr^−1^. A complicating factor is that at D_2_ 3 hr^−1^, 4 hr^−1^, and 6 hr^−1^, the outlet phage titres P_2_ were 3.8 × 10^8^ PFU/mL, 2.7 × 10^7^ PFU/mL, and 2 × 10^6^ PFU/mL, respectively. The phage amplification kinetics are seen to follow the mass action law (Figure 4b); hence, at the higher phage titres of 3.8 × 10^8^ PFU/mL, phage amplification in R3 was considerably faster and was over in less than one hour (see Table S3), whereas at D_2_ 6 hr^−1^, the considerably lower phage titres entering R3 meant that the amplification process took significantly longer (~3 hours to complete). The resulting differences in the final phage titres of ~2 × 10^11^ PFU/mL at D_2_ 3 hr^−1^ and ~6 × 10^10^ PFU/mL at D_2_ 6 hr^−1^ (*p* < 0.05 using a two-sample t-test, 95% confidence interval for the difference in means was 1.3 × 10^11^–2.1 × 10^11^ PFU/mL) may potentially be attributed to the host bacteria physiology changing in R3 over the 3-hr period that was needed to complete phage amplification. This point requires further investigation in the future.

Comparing phage titres for R1 dilution rates of 0.4 hr^−1^, 0.5 hr^−1^, and 0.6 hr^−1^ on phage production rates in R2 operating at a dilution rate D_2_ of 6 hr^−1^ (Figure 4) showed an increase in phage titres from 6.8 × 10^4^ PFU/mL (D_1_ 0.6 hr^−1^) to 1.7 × 10^5^ PFU/mL (D_1_ 0.4 hr^−1^) and the highest value at 2.1 × 10^6^ PFU/mL operating R1 at the optimum dilution rate D_1_ of 0.5 hr^−1^. This corresponds to phage productivity values of 4.1 × 10^5^ PFU/mL. hr (D_1_ 0.6 hr^−1^), 1 × 10^6^ PFU/mL. hr (D_1_ 0.4 hr^−1^), and 1.3 × 10^7^ PFU/mL. hr (D_1_ 0.5 hr^−1^), which were indicative of the different phage amplification rates. It has previously been shown that the rate of phage release increases with the increasing bacterial growth rate due to higher rates of synthesis and the assembly of phage components, and this is dependent on the content of the protein-synthesizing system. This manifests through a reduction in the latency period and an increase in the phage burst size [35]. The chemostat system that was used here (reactors R1 and R2 operating in series) is a versatile tool allowing control over the host physiology by controlling the dilution rate and thereby the host cell characteristics, including the size, age, rates of metabolism, chromosome replication, and time of lysis. Cells growing in richer media have been shown to have shorter doubling times. The corresponding rates of phage adsorption have been shown to increase substantially in such cases in comparison with the same strain grown in restricted media, in which the rates of phage development and phage lysis are dependent on the host bacteria growth rates, and an order of magnitude difference in burst sizes was reported [35].

In this study, we used a completely synthetic medium with glucose as the only sugar source as the limiting substrate. Previous studies using chemostats to investigate the effect of host physiology have used complex ill-defined media where it was unclear which limiting factor affected the host organism growth rate, and therefore influenced the parameters affecting phage amplification [39]. Using the value of the maximum specific growth rate (µ_m_ = 1 hr^−1^) results in a doubling time of ~42 min. Hence, using D_1_ values of 0.4 hr^−1^, 0.5 hr^−1^, and 0.6 hr^−1^ yields ~3.5, ~2.9, and ~2.4 cell divisions, respectively, for the three cases. Different dilution rates may result in considerably different age distributions of the host cells in the reactor. The chemostat provides a means to obtaining steady-state cultures with a synchronized narrow distribution of cell ages, and may therefore allow tuning of the age distribution of cells undergoing cell division. Cells that might be more mature and nearing division (e.g., for the case D_1_ 0.5 hr^−1^) have previously been shown to yield higher burst sizes and showed shorter lag times, and this was shown to correlate with the availability of intracellular resources [40]. The infection of mature cells closer to cell division that contain nucleic acid precursors and translational machinery for efficient phage assembly may yield higher phage burst sizes and shorter lag times, thereby resulting in a significant increase in the phage productivity that was observed in the present study [37]. Mature cells that are closer to cell division harbour higher levels of ribosomes, polymerases, and other molecules that are necessary for the transcription of early, middle, and late-stage phage proteins [41]. The expression levels of the early transcripts that are necessary for lytic phage development have been shown to be affected in minimal media as a host response to physiological conditions [42].

In a previous study using *E. coli* T4 phages and dilution rates between 0.033–0.3 hr^−1^, considerable differences in burst sizes were observed ranging between 1–15, and lag times decreasing from 3 hr to ~1 hr with increasing dilution rates [38]. The study employed minimal media with a high inlet concentration of glucose (10 g L^−1^) as the only sugar source. However, the range of dilution rates was considerably lower in comparison to those used in the present study, and no measurements of sugar concentration in the reactor were reported in the paper. Given the long residence times in the reactor, the host bacteria were in the late exponential growth to almost the stationary phase. In the present study, the dilution rates in the host bioreactor were between 0.4–0.6 hr^−1^, and the host bacteria were in the early to mid-exponential growth phase, with considerably shorter doubling times around ~40 min compared with 21 hr. Monod kinetics fitted the experimental data well (µ_m_ = 1 hr^−1^ and K_m_ = 1.5 g L^−1^), which allowed an estimation of the host bacteria growth rates (Figure 2b). The glucose substrate consumption rates were three-fold higher when the dilution rates were 0.5 hr^−1^ and 0.6 hr^−1^ compared with the glucose consumption rate at 0.4 hr^−1^. This indicates higher metabolic rates for the host bacteria in the early to mid-growth stages such as those growing at the dilution rate of 0.5 hr^−1^. *E coli* T7 phages grown in a chemostat using rich LB medium and high dilution rates in excess of 1 hr^−1^ resulted in the burst size (b ~ 60) almost doubling in comparison with measured burst size values of ~30 at a dilution rate of 0.5 hr^−1^ [37]. The authors of this paper not only evaluated the effect of dilution rates on burst size, but also the time required by the host cell to produce the first phage progeny (eclipse time) and the intracellular rate of phage progeny production (rise rate), and showed that an increase in the rise rate correlated with the bacterial host growth rate [37].

Investigation was undertaken as to whether the lag times, burst sizes, and phage adsorption rates were indeed different for host cells under steady-state conditions for D_1_ 0.4 hr^−1^, 0.5 hr^−1^, and 0.6 hr^−1^. The experimentally measured phage adsorption binding rates and lag times were not found to be significantly different for the three dilution rates (see Supplementary Information, Figure S2). The burst sizes were significantly different for the three dilution rates, with average burst sizes varying from ~10 for 0.6 hr^−1^, ~20 for 0.4 hr^−1^, and as high as ~40 for the host cells that were subjected to the dilution rate of 0.5 hr^−1^ (see Supplementary Information, Figure S1). Hence, the faster growth of host cells that may be nearing cell division did seem to support increased phage productivity, as observed in our results (Figure 4).

**Figure 6:**
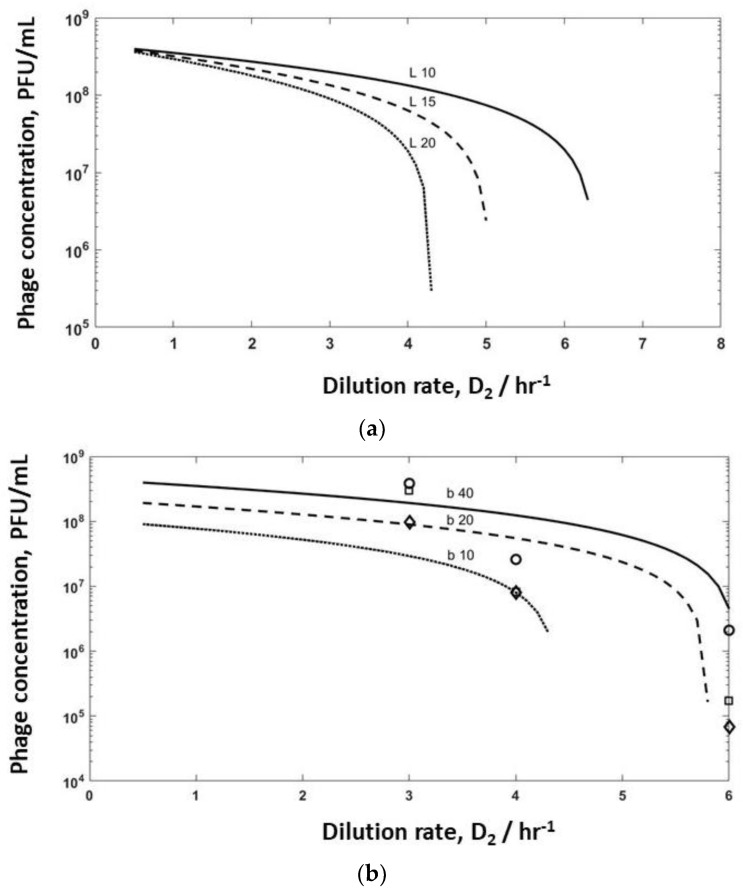
Simulation results showing the effect of (**a**) lag time on phage washout; a burst size of 40 and adsorption rate of 3.6 × 10^−11^ L hr^−1^ was used for the simulations; (**b**) burst size on phage titres and washout of phages in reactor R2: the dotted line represents the results of the numerical simulations using adsorption rates of 9 × 10^−11^ L hr^−1^ and a burst size of 10, the dashed line represents the results of the numerical simulations using adsorption rates of 7 × 10^−11^ L hr^−1^ and a burst size of 20, the solid line represents the results of the numerical simulations using adsorption rates of 3.6 × 10^−11^ L hr^−1^ and a burst size of 40. Open squares (□) correspond to phages produced in R2 with R1 operated at D_1_= 0.4 hr^−1^; open round circles (o) correspond to phages produced in R2 with R1 operated at D_1_= 0.5 hr^−1^; open diamonds (◊) correspond to phages produced in R2 with R1 operated at D_1_= 0.6 hr^−1^. The other simulation parameters that were used for all of the simulations were: C_1_ = 1.1 × 10^10^ CFU L^−1^, S_1_ = 1.5 g L^−1^ and a lag time of 10 min.

Model simulations were undertaken for bioreactor R2 to evaluate the effect of varying burst sizes (b varied in the range of 10–40, and lag times varied between 10–20 min) to identify washout conditions on phage production (Figure 6). Increasing the lag time from 10 min to 15 min had a significant effect on phage titres leaving R2, with washout occurring if the lag time was increased to 20 min (Figure 6a) at high dilution rates of D_2_ ~6 hr^−1^. At low dilution rates, the phage titres converged, and the effect of the differences in lag times was less pronounced. Equating r_2_ = D_2_P_2_ = δbC_2_P_2_ allowed the in situ determination of the adsorption rates at dilution rates of D_1_ 0.4 hr^−1^ (δ = 1 × 10^−11^ L hr^−1^), D_1_ 0.5 hr^−1^ (δ = 3.6 × 10^−11^ L hr^−1^), and D_1_ 0.6 hr^−1^ (δ = 9 × 10^−11^ L hr^−1^), respectively. Using these adsorption rates and a lag time of 10 min, burst size was varied (b = 10, 20, and 40) when using the mathematical model describing the dynamics of R2 (Equation (9)). Experimental data (Figure 4a) have been overlayed on the same plot (Figure 6b). The simulation results show a similar trend to the experimental data, with small variations in burst size and adsorption rates having a significant impact on the predicted values of the phage titres in R2 and phage washout as a function of the dilution rate D_2_ (Figure 6b). It is unclear at present why the *in situ* fitted adsorption rate values were significantly lower than those that were measured experimentally (Figure S2).

**Figure 7:**
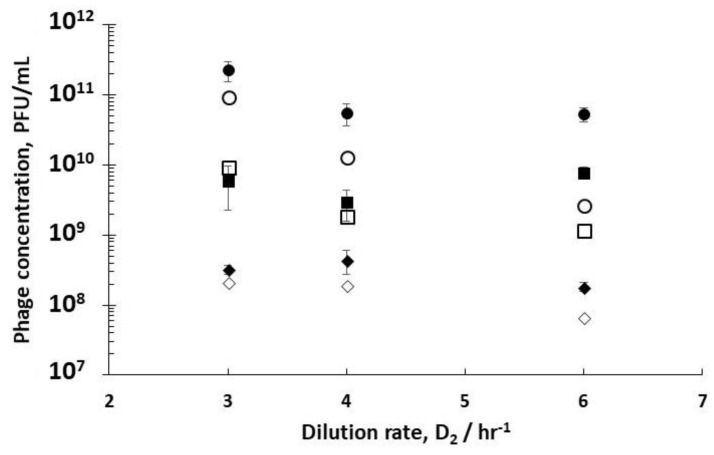
Simulation results showing modelling fit of final phage titres in reactor R3 using output conditions from reactor R2 as inlet conditions for the semi-batch operation of R3. Filled squares (■) correspond to experimental data for phages infected in R2 when R1 was operated at D_1_= 0.4 hr^−1^; open squares (□) correspond to simulation results at D_1_= 0.4 hr^−1^ using a burst size of 20 and a lag time of 60 min. The inlet conditions that were used for simulations were: C_2_ = 4 × 10^10^ CFU L^−1^, P_2_ [3.0 × 10^11^, 8.3 × 10^9^, 1.7 × 10^8^], PFU l^−1^ corresponding to D_2_ [3, 4, 6], S_2_ = 1.1 g L^−1^. Filled round circles (●) correspond to experimental data for phages infected in R2 when R1 was operated at D_1_= 0.5 hr^−1^; open round circles (ο) correspond to simulation results at D_1_= 0.5 hr^−1^ using a burst size of 100 and a lag time of 60 min. The inlet conditions that were used for simulations were: C_2_ = 1 × 10^10^ CFU L^−1^, P_2_ [3.8 × 10^11^, 2.6 × 10^10^, 2.1 × 10^9^], PFU l^−1^ corresponding to D_2_ [3, 4, 6], S_2_ = 1.5 g L^−1^. Filled diamonds (♦) correspond to experimental data for phages infected in R2 when R1 was operated at D_1_= 0.6 hr^−1^; open diamonds (◊) correspond to simulation results at D_1_= 0.6 hr^−1^ using a burst size of 10 and a lag time of 60 min. The inlet conditions that were used for simulations were: C_2_ = 3.7 × 10^9^ CFU L^−1^, P_2_ [1 × 10^10^, 8 × 10^9^, 7 × 10^7^], PFU l^−1^ corresponding to D_2_ [3, 4, 6], S_2_ = 2.3 g L^−1^. The other simulation parameters that were used for all of the simulations were: δ = 3.6 × 10^−9^ L hr^−1^, and the semi-batch reactor inlet flow rate that was used was 0.25 L hr^−1^.

**Figure 8:**
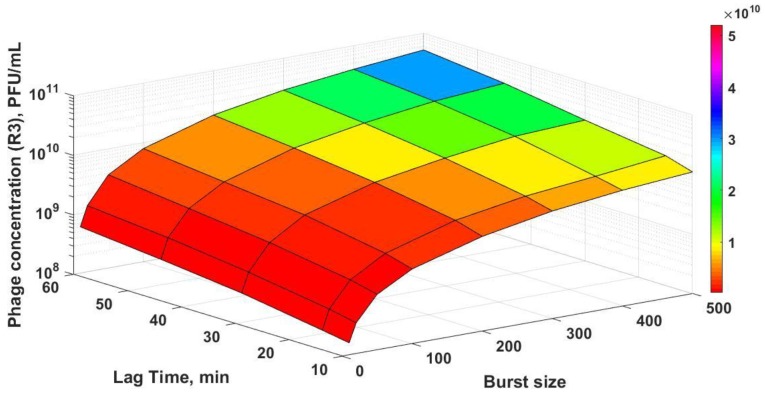
Surface plot showing simulation results of final phage titres in reactor R3 as a function of burst sizes [10, 20, 50, 100, 200, 300, 400, and 500] and lag times [10, 15, 30, 45, and 60] min. The other fixed simulation parameters were: C_2_ = 1 × 10^10^ CFU L^−1^, P_2_ 1 × 10^10^ PFU l^−1^, S_2_ = 1.5 g L^−1^, δ = 3.6 × 10^−9^ L hr^−1^, and the semi-batch reactor inlet flow rate used was 0.25 L hr^−1^.

Using experimental inlet conditions for the different dilution rate combinations of D_1_ and D_2_ that were evaluated in the study, simulations for R3 were carried out by numerically solving Equations (10)–(12). The model predictions were considerably lower in comparison with experimentally measured phage titres in R3 (see Supplementary Information, Figure S3) when the experimentally measured burst sizes (10–40), lag times (~10 min), and adsorption rates were used. Better correspondence between the experimental data and results of numerical simulations could be achieved by varying the values of the burst sizes and lag times (Figure 7). Simulation results were closer to experimental data at lower dilution rates of D_2_ 3 hr^−1^ compared with the results at D_2_ 6 hr^−1^, which suggests that model parameters may need to be varied depending on the dynamics of the unsteady process in R3. Phage amplification in R3 was rapid (phage amplification was complete within an hour with no significant change in sugar concentration) at D_2_ 3 hr^−1^, whereas it took around two hours at D_2_ 6 hr^−1^, and the sugar concentration in the reactor changed substantially in this period (see Supplementary Information, Table S3). Plotting the simulation results as a surface plot (Figure 8) shows how the final phage titres in R3 are affected as a function of both the burst size and lag times, with final phage titres increasing with both burst size and lag times. In the unsteady-state of semi-batch reactor R3, the host cell physiology and therefore the phage infection parameters that were evaluated in R2 may not remain the same if amplification occurs over several hours post-R2 (e.g., the sugar concentration in R3 was found to vary over time, which would affect the host bacteria growth rates and phage production). This may explain the discrepancies between the results of the numerical simulations and the experimental data. R2 was operated under controlled steady-state conditions; therefore, the parameters that were evaluated were carefully controlled. However, this was not the case in reactor R3. Although it was not the focus of the present study, future experimental work needs to be carried out in order to investigate the phage–bacterium dynamics for reactor R3 through undertaking dynamic simulations and incorporating the effects of variable infection parameters as a function of the bacterial growth rate. This would allow a more systematic optimization of Reactor 3 for the process outlined here [30].

## 5. Conclusions

The continuous production of high titres of phages was shown to be reliably achievable using two continuous stirred tank bioreactors connected in series and a final stirred tank operated in semi-batch mode to finish the infection process. The first bioreactor allowed the steady-state propagation of host bacteria using a fully synthetic medium, and host bacterial growth was decoupled from the phage production reactor downstream of it, thereby suppressing phage-related host mutation. Manipulation of the host reactor dilution rates allowed control over the physiological state of the bacterial host, which was fed to the phage infection reactor. This permitted an evaluation of the optimum conditions that were conducive for high intracellular phage production. A continuous supply of high concentrations of early to mid-growth phase readily infected bacteria was maintained to the phage production reactor. Manipulating the dilution rates through the two bioreactors (using different working volumes) allowed control over the process operating conditions, including the steady-state bacterial growth rates and cell concentration in reactor 1, the phage concentration and infection dynamics in reactor 2, the conversion of the growth-limiting substrate (glucose), and the overall phage productivity of the process.

Operating reactor 1 at high dilution rates allowed optimum exponentially growing bacteria to be fed to the phage amplification reactor 2, resulting in much higher phage production rates compared with bacteria in the late exponential growth phase. Mathematical modelling of the dynamics of the process allowed parameter sensitivity evaluation and insights gained into the factors affecting the phage production process. The host bacterium physiology was found to strongly influence the phage burst sizes, thereby affecting the productivity of the overall process.

## Supplementary Materials

The following are available online at http://www.mdpi.com/1999-4915/10/10/537/s1, Figure S1: *E. coli* T3 phage development (one step growth), Figure S2: Rates of adsorption of *E. coli* T3 phage to exponentially growing host bacteria in chemostat R1, Figure S3: Simulation results showing modelling fit of final phage titres in R3, Table S1: Composition of synthetic medium, Table S2: Summary of experimental runs, Table S3: Phage titre P_3_ amplification in bioreactor R3.
