# Supplementary material for: High Throughput Manufacturing of Bacteriophages Using Continuous Stirred Tank Bioreactors Connected in Series to Ensure Optimum Host Bacteria Physiology for Phage Production
Source: Viruses. 2018 Oct 1;10(10):537. doi: 10.3390/v10100537 (PMC6213498; doi:10.3390/v10100537)
Supplement: Supplementary file 1 [file viruses-10-00537-s001.pdf]

Supplementary Information.

Table S1. Composition of synthetic medium.

Name	Working concentration	Sterilization method
**$MgSO_4$**	0.586 g L^{-1}	121 °C autoclave
**Glucose**	2.94 g L^{-1}	Filtration (0.22 μm)
**$Na_2MoO_4 \cdot H_2O$**	2.1 mg L^{-1}	121 °C autoclave
**Trace elements.**	2.5mg L^{-1} $CoCl_2{\cdot}6H_2O$	121 °C autoclave.
.	15 mg L^{-1} $MnCl_2{\cdot}4H_2O$	.
.	1.5 mg L^{-1} $CuCl_2{\cdot}2H_2O$	.
.	3 mg L^{-1} H_3BO_3	.
.	33.8 mg L^{-1} $Zn(CH_3COO)_2{\cdot}2H_2O$	.
	14.10 mg L^{-1} Titriplex III	
**$(NH_4)_2HPO_4$**	4 g L^{-1}	121 °C autoclave
**KH_2PO_4**	13.3 g L^{-1}	121 °C autoclave
**Citric Acid**	1.5542 g L^{-1}	121 °C autoclave
**Fe(III) citrate**	0.1008 g L^{-1}	121 °C autoclave
**Trizma Base**	To bring pH to 7	121 °C autoclave

Table S2. Summary of experimental runs. (a) changing dilution rate in host propagation reactor (R1) to control host bacterium physiology; (b) changing different dilution rates in R1 to affect host bacterium physiology and investigate the effect of this on phage productivity in reactor (R2) with R2 operated at a constant dilution rate 4 hr^{-1}; (c) experiment runs to investigate the effect of constant host physiology (dilution rates in R1 fixed at either 0.4 hr^{-1}, 0.5 hr^{-1} or 0.6 hr^{-1}) and changing dilution rates in R2 to study effect on phage titres in R2.

(**a**).

Dilution rate R1, hr^{-1}	Flow rate mL hr^{-1}	Volume R1 mL	Residence time in R1, hr
0.1	50	500	10
0.2	100	500	5
0.3	150	500	3.33
0.4	200	500	2.5
0.5	250	500	2
0.6	300	500	1.6

(**b**).

Dilution rate R1 (hr^{-1})	Dilution rate R2 (hr^{-1})	Flow rate (mL/hr)	Volume R2 (mL)	Residence time in R2 (min)
0.1	4	50	12.5	15
0.2	4	100	25	15
0.3	4	150	37.5	15
0.4	4	200	50	15
0.5	4	250	62.5	15
0.6	4	300	75	15

(**c**).

Dilution rate R1 (hr^{-1})	Dilution rate R2 (hr^{-1})	Flow rate (mL/hr)	Volume R2 (mL)	Residence time in R2 (min)
0.4	3	200	67	20

0.4	4	200	50	15
0.4	6	200	33.3	10
0.5	3	250	83.3	20
0.5	4	250	62.5	15
0.5	6	250	42	10
0.6	3	300	100	20
0.6	4	300	75	15
0.6	6	300	50	10

Table S3. Phage titre P_3 amplification in bioreactor R3, D_1 0.5 hr^{-1}, $C_2 = 1.1 \times 10^7$ CFU mL^{-1}. Time 0 hr represents phage titre inlet conditions to R3.

	**D_2 3 hr^{-1}**		**D_2 6 hr^{-1}**	
Time, hr	Glucose, g L^{-1}	P_3, PFU mL^{-1}	Glucose, g L^{-1}	P_3, PFU mL^{-1}
0	0.72	3.8×10^8	0.86	2.1×10^6
1	0.70	1.5×10^{11}	0.64	7×10^8
2	0.64	2.7×10^{11}	0.20	2.3×10^{10}

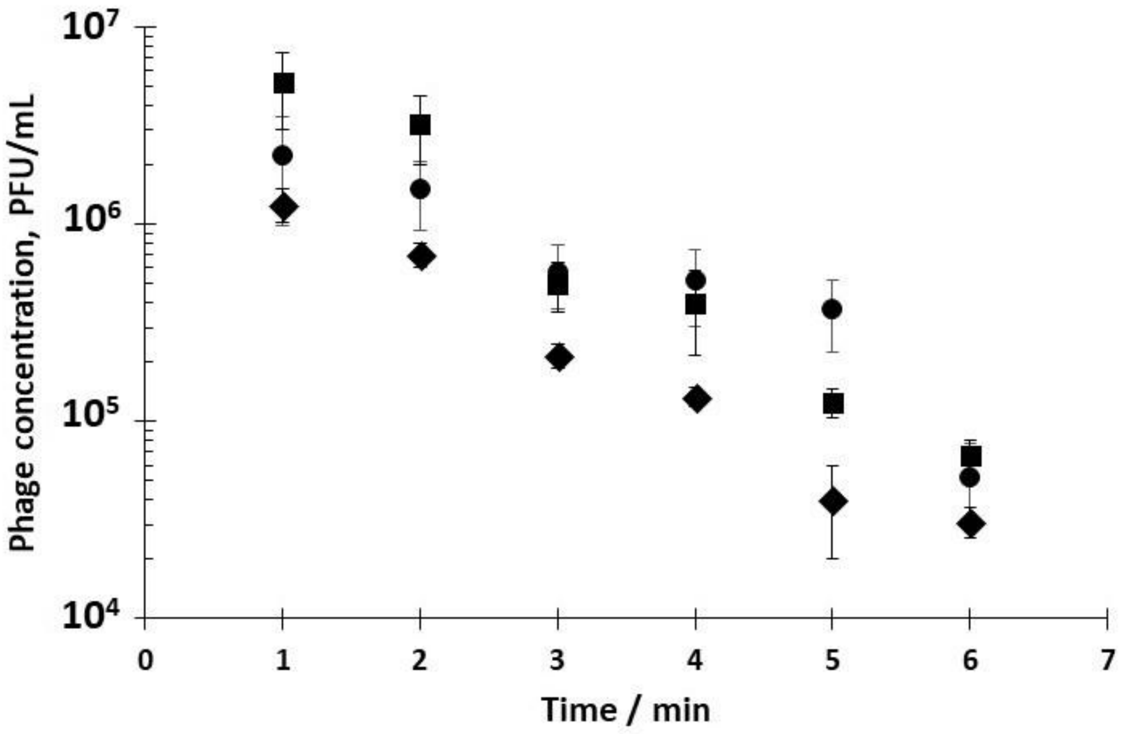

Figure S1. Rates of adsorption of *E. coli* T3 phage to exponentially growing host bacteria in chemostat R1 at dilution rates 0.4 hr^{-1} (squares), phage adsorption rate estimated using linear least squares fit (δ) 1.8×10^{-9} L hr^{-1} (r^2 0.964), 0.5 hr^{-1} (circles) phage adsorption rate (δ) 3.6×10^{-9} L hr^{-1} (r^2 0.877), and 0.6 hr^{-1} (diamonds) phage adsorption rate (δ) 9×10^{-9} L hr^{-1} (r^2 0.868). Error bars represent one standard deviation.

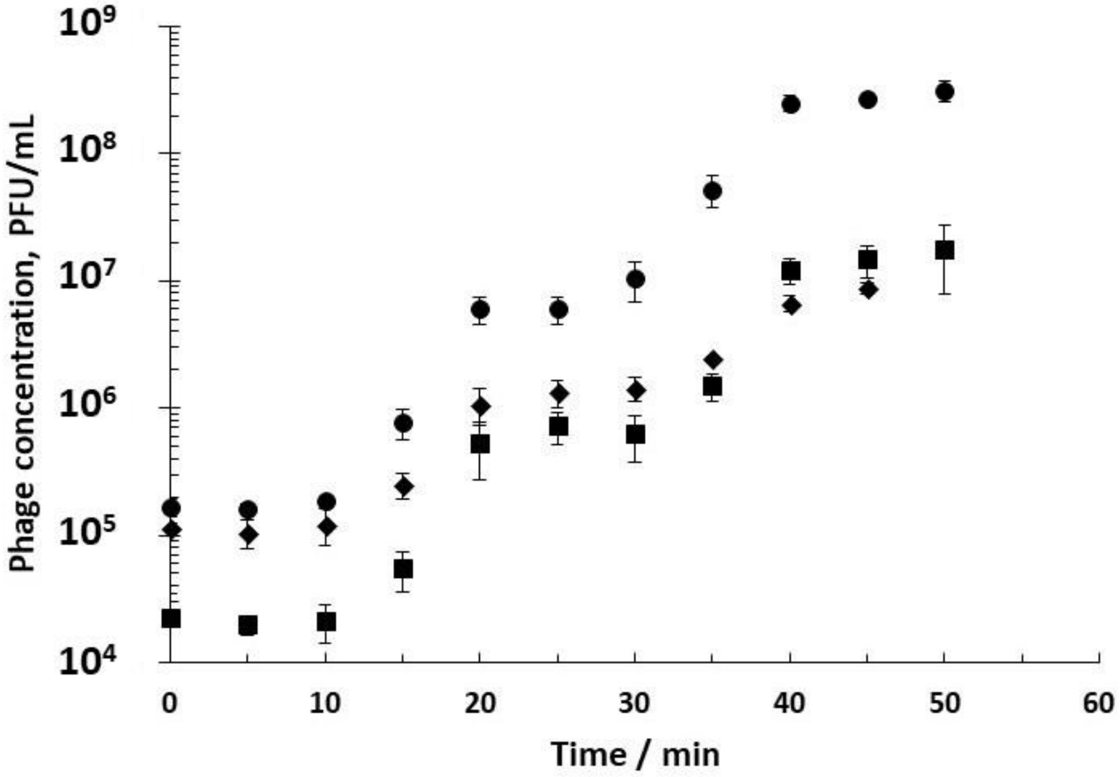

Figure S2. *E. coli* T3 phage development (one step growth) in single infections of steady-state exponentially growing *E. coli* host bacteria in chemostat R1 at dilution rates 0.4 hr^{-1} (squares), burst size ~ 20, 0.5 hr^{-1} (circles) burst size ~ 40 and 0.6 hr^{-1} (diamonds) burst size ~ 10. Error bars represent one standard deviation.

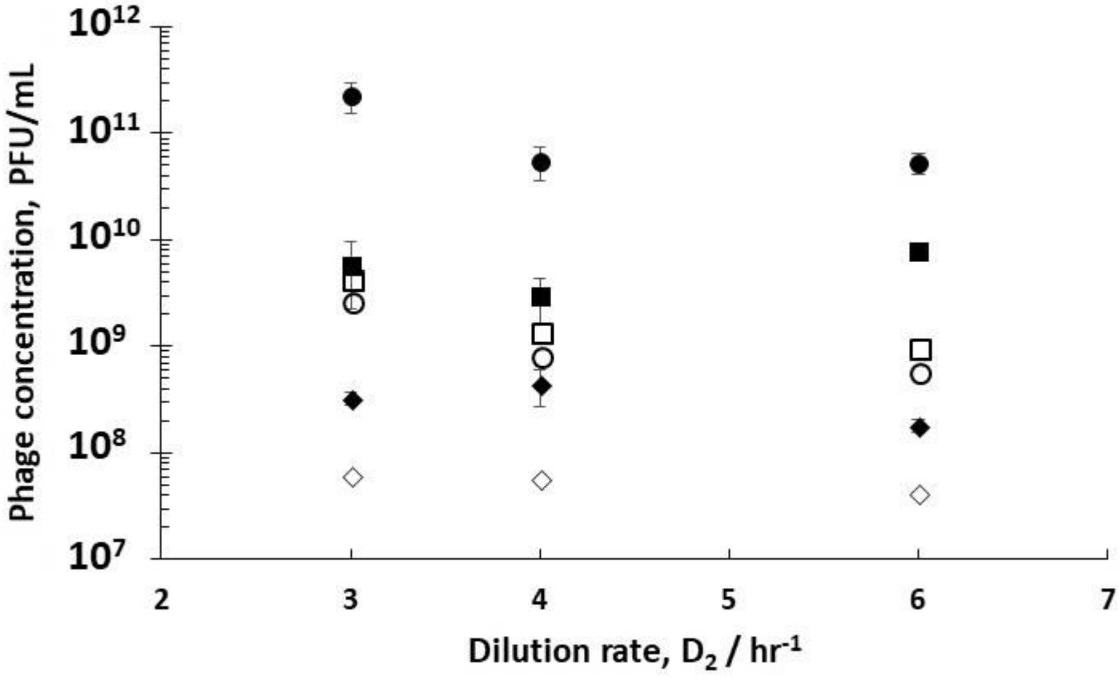

Figure S3. Simulation results showing modelling fit of final phage titres in reactor R3 using output conditions from reactor R2 as inlet conditions for semi-batch operation of R3. Filled squares (■)

correspond to phages infected in R2 when R1 was operated at D_1= 0.4 hr^{-1}, Open squares (□) correspond to simulation results at D_1= 0.4 hr^{-1} using a burst size of 20 and lag time of 10 min. Inlet conditions used for simulations were: $C_2 = 4 \times 10^{10}$ CFU L^{-1}, P_2 [3.0×10^{11}, 8.3×10^{9}, 1.7×10^{8}] PFU l^{-1} corresponding to D_2 [3, 4, 6], S_2 = 1.1 g L^{-1}. Filled round circles (●) correspond to phages infected in R2 when R1 was operated at D_1= 0.5 hr^{-1}; Open round circles (o) correspond to simulation results at D_1= 0.5 hr^{-1} using a burst size of 40 and lag time of 10 min. Inlet conditions used for simulations were: C_2 = 1×10^{10} CFU L^{-1}, P_2 [3.8×10^{11}, 2.6×10^{10}, 2.1×10^{9}] PFU l^{-1} corresponding to D_2 [3, 4, 6], S_2 = 1.5 g L^{-1}. Filled diamonds (♦) correspond to phages infected in R2 when R1 was operated at D_1= 0.6 hr^{-1}; Open diamonds (◊) correspond to simulation results at D_1= 0.6 hr^{-1} using a burst size of 10 and lag time of 10 min. Inlet conditions used for simulations were: $C_2 = 3.7 \times 10^{9}$ CFU L^{-1}, P_2 [1×10^{10}, 8×10^{9}, 7×10^{7}] PFU l^{-1} corresponding to D_2 [3, 4, 6], S_2 = 2.3 g L^{-1}. Other simulation parameters used for all simulations were: $\delta = 3.6 \times 10^{-9}$ L hr^{-1} and the semi-batch reactor inlet flow rate used was 0.25 L hr^{-1}.
